# Bis[4-(dimethyl­amino)pyridinium] tetra­bromidodiphenyl­plumbate(IV)

**DOI:** 10.1107/S1600536808027530

**Published:** 2008-09-06

**Authors:** Kong Mun Lo, Seik Weng Ng

**Affiliations:** a Department of Chemistry, University of Malaya, 50603 Kuala Lumpur, Malaysia

## Abstract

The Pb^IV^ atom of the anion of the title salt, (C_7_H_11_N_2_)_2_[PbBr_4_(C_6_H_5_)_2_], is situated on a crystallographic center of inversion and exhibits a tetra­gonally compressed octa­hedral coordination. One of the two independent Br atoms acts as a hydrogen-bond acceptor towards the NH group of the cation.

## Related literature

For the structure of isostructural bis­(4-dimethyl­amino­pyridinium) tetra­bromidodiphenyl­stannate, see: Yap *et al.* (2008 ▶).
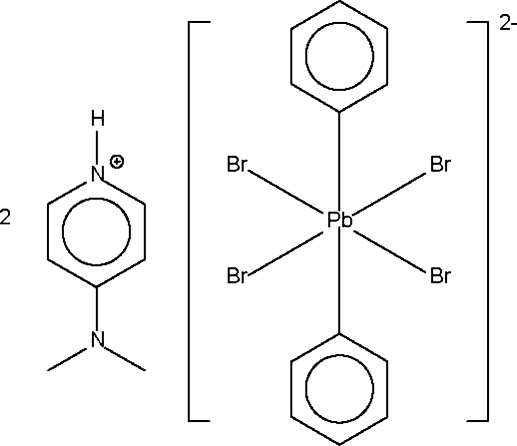


## Experimental

### 

#### Crystal data

(C_7_H_11_N_2_)_2_[PbBr_4_(C_6_H_5_)_2_]
*M* *_r_* = 927.39
Monoclinic, 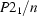

*a* = 9.4994 (8) Å
*b* = 13.882 (1) Å
*c* = 10.991 (1) Å
β = 92.998 (1)°
*V* = 1447.3 (2) Å^3^
*Z* = 2
Mo *K*α radiation
μ = 11.37 mm^−1^
*T* = 100 (2) K
0.22 × 0.08 × 0.06 mm

#### Data collection

Bruker SMART APEX diffractometer
Absorption correction: multi-scan (*SADABS*; Sheldrick, 1996 ▶) *T*_min_ = 0.246, *T*_max_ = 0.549 (expected range = 0.226–0.505)
8227 measured reflections
3309 independent reflections
2879 reflections with *I* > 2σ(*I*)
*R*_int_ = 0.029

#### Refinement

*R*[*F* ^2^ > 2σ(*F* ^2^)] = 0.029
*wR*(*F* ^2^) = 0.074
*S* = 1.03
3309 reflections
162 parameters
H-atom parameters constrained
Δρ_max_ = 1.21 e Å^−3^
Δρ_min_ = −1.70 e Å^−3^

### 

Data collection: *APEX2* (Bruker, 2007 ▶); cell refinement: *SAINT* (Bruker, 2007 ▶); data reduction: *SAINT*; program(s) used to solve structure: *SHELXS97* (Sheldrick, 2008 ▶); program(s) used to refine structure: *SHELXL97* (Sheldrick, 2008 ▶); molecular graphics: *X-SEED* (Barbour, 2001 ▶); software used to prepare material for publication: *publCIF* (Westrip, 2008 ▶).

## Supplementary Material

Crystal structure: contains datablocks global, I. DOI: 10.1107/S1600536808027530/im2080sup1.cif

Structure factors: contains datablocks I. DOI: 10.1107/S1600536808027530/im2080Isup2.hkl

Additional supplementary materials:  crystallographic information; 3D view; checkCIF report

## Figures and Tables

**Table 1:** Selected bond lengths (Å)

Pb1—C1	2.190 (5)
Pb1—Br1	2.8516 (5)
Pb1—Br2	2.8897 (5)

**Table 2:** Hydrogen-bond geometry (Å, °)

*D*—H⋯*A*	*D*—H	H⋯*A*	*D*⋯*A*	*D*—H⋯*A*
N2—H2*n*⋯Br1	0.88	2.52	3.254 (4)	142

**Fig. 1.**
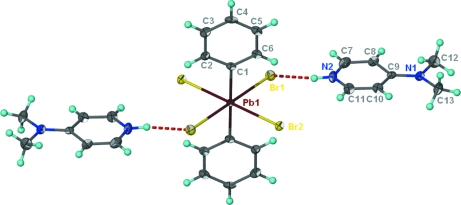
Thermal ellipsoid plot (Barbour, 2001) plot of (C7H11N)2 [PbBr4(C6H5)2] at the 70% probability level. Hydrogen atoms are drawn as spheres of arbitrary radius.

## supplementary crystallographic information

### Comment

In an earlier study, the tin-alkyl and one tin-aryl bond of an alkyltriphenyltin compound could be cleaved by 4-dimethylaminopyridine hydrobromide perbromide to form bis(4-dimethylaminopyridinium) tetrabromidodiphenylstannate (Yap *et al.*, 2008). In the present study, the organic reagent similarly cleaves two lead-carbon bonds to afford the corresponding plumbate (Scheme I, Fig. 1). The two compounds are isostructural.

### Experimental

Tetraphenyllead (1.55 g, 3 mmol) and 4-dimethylaminopyridinium hydrobromide perbromide (1.1 g, 3 mmol) were heated in chloroform (100 ml) for 3 h. The filtered solution when allowed to evaporate yielded large colorless crystals.

### Refinement

Carbon-bound H-atoms were placed in calculated positions (C—H 0.95 to 0.98 Å) and were included in the refinement in the riding model approximation, with *U*(H) set to 1.2 to 1.5*U*_eq_(C). The ammonium H atom was similarly constrained (N–H 0.88 Å).

The difference Fourier map had large peaks/deep holes near the lead atom.

### Crystal data

**Table:**

(C_7_H_11_N_2_)_2_[PbBr_4_(C_6_H_5_)_2_]	*F*(000) = 876
*M**_r_* = 927.39	*D*_x_ = 2.128 Mg m^−^^3^
Monoclinic, *P*2_1_/*n*	Mo *K*α radiation, λ = 0.71073 Å
Hall symbol: -P 2yn	Cell parameters from 3449 reflections
*a* = 9.4994 (8) Å	θ = 2.3–28.3°
*b* = 13.882 (1) Å	µ = 11.37 mm^−^^1^
*c* = 10.991 (1) Å	*T* = 100 K
β = 92.998 (1)°	Prism, colorless
*V* = 1447.3 (2) Å^3^	0.22 × 0.08 × 0.06 mm
*Z* = 2	

### Data collection

**Table:**

Bruker SMART APEX diffractometer	3309 independent reflections
Radiation source: fine-focus sealed tube	2879 reflections with *I* > 2σ(*I*)
graphite	*R*_int_ = 0.029
ω scans	θ_max_ = 27.5°, θ_min_ = 2.4°
Absorption correction: multi-scan (SADABS; Sheldrick, 1996)	*h* = −12→12
*T*_min_ = 0.246, *T*_max_ = 0.549	*k* = −17→18
8227 measured reflections	*l* = −14→14

### Refinement

**Table:**

Refinement on *F*^2^	Primary atom site location: structure-invariant direct methods
Least-squares matrix: full	Secondary atom site location: difference Fourier map
*R*[*F*^2^ > 2σ(*F*^2^)] = 0.029	Hydrogen site location: inferred from neighbouring sites
*wR*(*F*^2^) = 0.074	H-atom parameters constrained
*S* = 1.03	*w* = 1/[σ^2^(*F*_o_^2^) + (0.0374*P*)^2^ + 3.9748*P*] where *P* = (*F*_o_^2^ + 2*F*_c_^2^)/3
3309 reflections	(Δ/σ)_max_ = 0.001
162 parameters	Δρ_max_ = 1.21 e Å^−^^3^
0 restraints	Δρ_min_ = −1.70 e Å^−^^3^

### Fractional atomic coordinates and isotropic or equivalent isotropic displacement parameters (Å^2^)

**Table:**

	*x*	*y*	*z*	*U*_iso_*/*U*_eq_	
Pb1	0.5000	0.5000	0.5000	0.00948 (8)	
Br1	0.55832 (5)	0.61261 (3)	0.29156 (5)	0.01773 (12)	
Br2	0.76243 (5)	0.40245 (3)	0.46044 (4)	0.01405 (11)	
N1	1.1998 (4)	0.5912 (3)	−0.0112 (4)	0.0149 (8)	
N2	0.8888 (4)	0.5868 (3)	0.2341 (4)	0.0195 (9)	
H2n	0.8188	0.5819	0.2831	0.023*	
C1	0.6145 (5)	0.6028 (3)	0.6196 (4)	0.0122 (9)	
C2	0.5516 (5)	0.6319 (3)	0.7245 (4)	0.0138 (9)	
H2	0.4624	0.6071	0.7443	0.017*	
C3	0.6230 (5)	0.6988 (3)	0.8006 (4)	0.0171 (10)	
H3	0.5821	0.7202	0.8728	0.021*	
C4	0.7539 (5)	0.7339 (3)	0.7703 (4)	0.0163 (10)	
H4	0.8019	0.7795	0.8219	0.020*	
C5	0.8145 (5)	0.7028 (3)	0.6654 (4)	0.0161 (10)	
H5	0.9041	0.7269	0.6455	0.019*	
C6	0.7450 (5)	0.6367 (3)	0.5894 (4)	0.0136 (9)	
H6	0.7864	0.6150	0.5175	0.016*	
C7	0.8774 (5)	0.6473 (4)	0.1383 (5)	0.0209 (11)	
H7	0.7961	0.6869	0.1276	0.025*	
C8	0.9792 (5)	0.6529 (3)	0.0567 (5)	0.0162 (10)	
H8	0.9691	0.6961	−0.0102	0.019*	
C9	1.1018 (5)	0.5936 (3)	0.0715 (4)	0.0115 (9)	
C10	1.1123 (5)	0.5358 (4)	0.1782 (4)	0.0164 (10)	
H10	1.1945	0.4981	0.1952	0.020*	
C11	1.0064 (5)	0.5337 (4)	0.2559 (5)	0.0189 (10)	
H11	1.0150	0.4944	0.3266	0.023*	
C12	1.1934 (6)	0.6536 (4)	−0.1184 (5)	0.0235 (12)	
H12A	1.0949	0.6696	−0.1405	0.035*	
H12B	1.2346	0.6201	−0.1865	0.035*	
H12C	1.2463	0.7129	−0.1001	0.035*	
C13	1.3293 (5)	0.5345 (4)	0.0087 (5)	0.0195 (10)	
H13A	1.3939	0.5682	0.0667	0.029*	
H13B	1.3744	0.5264	−0.0688	0.029*	
H13C	1.3061	0.4712	0.0416	0.029*	

### Atomic displacement parameters (Å^2^)

**Table:**

	*U*^11^	*U*^22^	*U*^33^	*U*^12^	*U*^13^	*U*^23^
Pb1	0.01089 (12)	0.01025 (12)	0.00716 (12)	−0.00156 (9)	−0.00091 (8)	−0.00108 (9)
Br1	0.0182 (2)	0.0185 (2)	0.0164 (2)	0.00011 (18)	0.00039 (19)	0.00211 (19)
Br2	0.0142 (2)	0.0158 (2)	0.0120 (2)	0.00186 (17)	−0.00032 (18)	−0.00038 (17)
N1	0.018 (2)	0.0146 (19)	0.012 (2)	0.0033 (16)	0.0018 (17)	0.0019 (16)
N2	0.018 (2)	0.025 (2)	0.016 (2)	−0.0001 (18)	0.0063 (18)	−0.0024 (18)
C1	0.014 (2)	0.011 (2)	0.011 (2)	−0.0021 (17)	−0.0035 (18)	0.0002 (17)
C2	0.015 (2)	0.013 (2)	0.014 (2)	0.0022 (18)	0.0000 (19)	0.0002 (18)
C3	0.022 (2)	0.018 (2)	0.011 (2)	0.003 (2)	−0.003 (2)	−0.0025 (19)
C4	0.023 (3)	0.009 (2)	0.016 (3)	−0.0022 (19)	−0.008 (2)	−0.0004 (18)
C5	0.016 (2)	0.016 (2)	0.016 (2)	−0.0037 (19)	−0.004 (2)	0.0035 (19)
C6	0.020 (2)	0.015 (2)	0.005 (2)	−0.0030 (19)	−0.0028 (18)	0.0005 (18)
C7	0.016 (2)	0.021 (3)	0.025 (3)	0.006 (2)	−0.005 (2)	−0.005 (2)
C8	0.019 (2)	0.016 (2)	0.013 (2)	0.0049 (19)	−0.002 (2)	0.0004 (19)
C9	0.013 (2)	0.011 (2)	0.011 (2)	−0.0004 (17)	−0.0021 (18)	−0.0026 (17)
C10	0.018 (2)	0.019 (2)	0.012 (2)	−0.001 (2)	−0.003 (2)	0.000 (2)
C11	0.019 (2)	0.023 (2)	0.015 (3)	−0.001 (2)	−0.001 (2)	−0.001 (2)
C12	0.030 (3)	0.023 (3)	0.018 (3)	0.004 (2)	0.003 (2)	0.008 (2)
C13	0.013 (2)	0.027 (3)	0.018 (3)	0.005 (2)	0.000 (2)	−0.003 (2)

### Geometric parameters (Å, °)

**Table:**

Pb1—C1	2.190 (5)	C4—H4	0.9500
Pb1—C1^i^	2.190 (5)	C5—C6	1.385 (6)
Pb1—Br1	2.8516 (5)	C5—H5	0.9500
Pb1—Br1^i^	2.8516 (5)	C6—H6	0.9500
Pb1—Br2^i^	2.8897 (5)	C7—C8	1.356 (7)
Pb1—Br2	2.8897 (5)	C7—H7	0.9500
N1—C9	1.335 (6)	C8—C9	1.428 (6)
N1—C13	1.467 (6)	C8—H8	0.9500
N1—C12	1.461 (6)	C9—C10	1.420 (7)
N2—C7	1.347 (7)	C10—C11	1.353 (7)
N2—C11	1.349 (7)	C10—H10	0.9500
N2—H2n	0.8800	C11—H11	0.9500
C1—C6	1.383 (6)	C12—H12A	0.9800
C1—C2	1.386 (6)	C12—H12B	0.9800
C2—C3	1.401 (7)	C12—H12C	0.9800
C2—H2	0.9500	C13—H13A	0.9800
C3—C4	1.392 (7)	C13—H13B	0.9800
C3—H3	0.9500	C13—H13C	0.9800
C4—C5	1.384 (7)		
			
C1—Pb1—C1^i^	180.00 (17)	C6—C5—C4	120.2 (4)
C1—Pb1—Br1	90.79 (12)	C6—C5—H5	119.9
C1^i^—Pb1—Br1	89.21 (12)	C4—C5—H5	119.9
C1—Pb1—Br1^i^	89.21 (12)	C1—C6—C5	119.1 (4)
C1^i^—Pb1—Br1^i^	90.79 (12)	C1—C6—H6	120.4
Br1—Pb1—Br1^i^	180.000 (16)	C5—C6—H6	120.4
C1—Pb1—Br2^i^	90.55 (12)	C8—C7—N2	121.5 (5)
C1^i^—Pb1—Br2^i^	89.45 (12)	C8—C7—H7	119.3
Br1—Pb1—Br2^i^	93.978 (14)	N2—C7—H7	119.3
Br1^i^—Pb1—Br2^i^	86.022 (14)	C7—C8—C9	119.8 (5)
C1—Pb1—Br2	89.45 (12)	C7—C8—H8	120.1
C1^i^—Pb1—Br2	90.55 (12)	C9—C8—H8	120.1
Br1—Pb1—Br2	86.022 (14)	N1—C9—C10	121.8 (4)
Br1^i^—Pb1—Br2	93.978 (14)	N1—C9—C8	122.0 (4)
Br2^i^—Pb1—Br2	180.0	C10—C9—C8	116.2 (4)
C9—N1—C13	121.4 (4)	C11—C10—C9	120.7 (5)
C9—N1—C12	122.2 (4)	C11—C10—H10	119.7
C13—N1—C12	115.9 (4)	C9—C10—H10	119.7
C7—N2—C11	120.7 (4)	C10—C11—N2	120.8 (5)
C7—N2—H2n	119.6	C10—C11—H11	119.6
C11—N2—H2n	119.6	N2—C11—H11	119.6
C6—C1—C2	122.1 (4)	N1—C12—H12A	109.5
C6—C1—Pb1	120.1 (3)	N1—C12—H12B	109.5
C2—C1—Pb1	117.8 (3)	H12A—C12—H12B	109.5
C1—C2—C3	118.2 (4)	N1—C12—H12C	109.5
C1—C2—H2	120.9	H12A—C12—H12C	109.5
C3—C2—H2	120.9	H12B—C12—H12C	109.5
C4—C3—C2	120.0 (4)	N1—C13—H13A	109.5
C4—C3—H3	120.0	N1—C13—H13B	109.5
C2—C3—H3	120.0	H13A—C13—H13B	109.5
C5—C4—C3	120.4 (4)	N1—C13—H13C	109.5
C5—C4—H4	119.8	H13A—C13—H13C	109.5
C3—C4—H4	119.8	H13B—C13—H13C	109.5
			
Br1—Pb1—C1—C6	−46.0 (4)	Pb1—C1—C6—C5	179.0 (3)
Br1^i^—Pb1—C1—C6	134.0 (4)	C4—C5—C6—C1	0.2 (7)
Br2^i^—Pb1—C1—C6	−140.0 (4)	C11—N2—C7—C8	3.9 (8)
Br2—Pb1—C1—C6	40.0 (4)	N2—C7—C8—C9	0.1 (8)
Br1—Pb1—C1—C2	133.8 (3)	C13—N1—C9—C10	−5.1 (7)
Br1^i^—Pb1—C1—C2	−46.2 (3)	C12—N1—C9—C10	−177.1 (5)
Br2^i^—Pb1—C1—C2	39.9 (3)	C13—N1—C9—C8	176.2 (4)
Br2—Pb1—C1—C2	−140.1 (3)	C12—N1—C9—C8	4.2 (7)
C6—C1—C2—C3	0.9 (7)	C7—C8—C9—N1	174.8 (5)
Pb1—C1—C2—C3	−179.0 (3)	C7—C8—C9—C10	−3.9 (7)
C1—C2—C3—C4	−0.3 (7)	N1—C9—C10—C11	−174.7 (5)
C2—C3—C4—C5	−0.2 (7)	C8—C9—C10—C11	4.0 (7)
C3—C4—C5—C6	0.3 (7)	C9—C10—C11—N2	−0.3 (8)
C2—C1—C6—C5	−0.8 (7)	C7—N2—C11—C10	−3.8 (8)

Symmetry codes: (i) −*x*+1, −*y*+1, −*z*+1.

### Hydrogen-bond geometry (Å, °)

**Table:**

*D*—H···*A*	*D*—H	H···*A*	*D*···*A*	*D*—H···*A*
N2—H2*n*···Br1	0.88	2.52	3.254 (4)	142
